# Epidemiological Society

**Published:** 1897-05-08

**Authors:** 


### EPIDEMIOLOGICAL SOCIETY.

To our minds the most interesting matter which Dr. Parkes brought forward in his paper on the Infectivity of Diphtheria was his demonstration of the increased frequency with which what he calls multiple cases occur in times of epidemic, that is cases in which several persons in the same house are attacked.

Many of the statistics which he gave went no doubt to strengthen the proof that school influence is one factor in the dissemination of the disease. But, after all, that is a matter which we may now almost take for granted; for indeed it would be strange if schools did not aid in the diffusion of a disease which is acknowledged to be spread by infection from patient to patient.

What is of interest is the proof given by Dr. Parkes' figures that, in the production of an epidemic, something is involved outside and beyond any mere increase in the chances of infection.

Comparing different periods, it was found that there were, in proportion, more than twice as many multiple cases when the disease was epidemic than when it was present only at its normal rate. Again, taking the multiple cases, it came out that the instances in which four or five persons were affected in the same house were proportionately twice as frequent during epidemic as during non-epidemic periods.

We have, then, the statistical demonstration that the tendency of the disease to spread at home is greater during some periods than during others; and it is interesting, also, to observe that, during these conditions of greater prevalence and greater tendency to spread, the case mortality of diphtheria is also greater than it is at other times, a matter in which this disease differs from scarlet fever, the prevalence and the fatality of which vary in almost inverse ratios.

When we consider how greatly our knowledge of diphtheria has increased in some directions, and how little in others, we cannot but think that the time is approaching when the field of observation should be shifted from the laboratory and the study to the homes in which the malady occurs. What we now want to know is what are the home conditions, what are the climatic conditions, and what are the conditions in regard to food, such as milk supply, which tend to render the disease virulent. This cannot, we fear, be found out by statistical inquiry. It is to patient investigation and comparison of individual groups of cases that we must look for an answer to these questions.

